# Intraoperative ventilatory leak predicts prolonged air leak after lung resection: A retrospective observational study

**DOI:** 10.1371/journal.pone.0187598

**Published:** 2017-11-09

**Authors:** Won Ho Kim, Hyung-Chul Lee, Ho-Geol Ryu, Hyun-Kyu Yoon, Chul-Woo Jung

**Affiliations:** Department of Anesthesiology and Pain Medicine, Seoul National University Hospital, Seoul, Republic of Korea; Peking University People's Hospital, CHINA

## Abstract

Prolonged air leak (PAL), defined as air leak more than 5 days after lung resection, has been associated with various adverse outcomes. However, studies on intraoperative risk factors for PAL are not sufficient. We investigated whether the intraoperative ventilatory leak (VL) can predict PAL. A retrospective study of 1060 patients with chest tubes after lung resection was conducted. Tidal volume data were retrieved from the electronic anesthesia records. Ventilatory leak (%) was calculated as [(inspiratory tidal volume—expiratory tidal volume)/ inspiratory tidal volume × 100] and was measured after restart of two-lung ventilation. Cox proportional hazards regression analysis was performed using VL as a predictor, and PAL as the dependent outcome. The odds ratio of the VL was then adjusted by adding possible risk factors including patient characteristics, pulmonary function and surgical factors. The incidence of PAL was 18.7%. VL >9.5% was a significant predictor of PAL in univariable analysis. VL remained significant as a predictor of PAL (1.59, 95% CI, 1.37–1.85, *P* <0.001) after adjusting for 7 additional risk factors including male gender, age >60 years, body mass index <21.5 kg/m^2^, forced expiratory volume in 1 sec <80%, thoracotomy, major lung resection, and one-lung ventilation time >2.1 hours. C-statistic of the prediction model was 0.80 (95% CI, 0.77–0.82). In conclusion, VL was a quantitative measure of intraoperative air leakage and an independent predictor of postoperative PAL. Monitoring VL during lung resection may be uselful in recommending additional surgical repair or use of adjuncts and thus, help reduce postoperative PAL.

## Introduction

Air leakage immediately after lung resection is common with an incidence of up to 60% and persists in 8% by postoperative day (POD) 4 [1,2]. Prolonged air leak (PAL) is defined as air leakage lasting beyond 5 days after routine lung resection surgery [1,3]. Due to its association with adverse clinical outcomes and the challenges in postoperative management, prediction and prevention of PAL has been an important issue after lung resection [1,3,4].

Predicting the occurrence of PAL has focused on the preoperative risk factors such as patient demographics, lung function, and preexisting lung disease [3,5,6]. Several intraoperative risk factors have also been studied [3], but intraoperative air leak, despite its potential association with postoperative air leak, has not been thoroughly investigated. Commonly performed intraoperative tests such as water-submersion test are less objective and can be biased [7,8].

In the current study, we measured the intraoperative air leakgeage, or ventilatory leak (VL), which was defined as the difference between inspiratory and expiratory tidal volumes measured during mechanical ventilation. We speculated that VL would be an objectivite and quantitative measure of air leakage during surgery, and thus, an independent predictor of PAL after lung resection. The purpose of this retrospective study was to identify the association between intraoperative VL and PAL after lung resection.

## Methods

This retrospective observational study was approved by the Institutional Review Board of Seoul National University Hospital (IRB number: H-1604-109-756) and was compliant to the STrengthening the Reporting of OBservational Studies in Epidemiology (STROBE) checklist (S1 Checklist). Informed consent was waived due to the retrospective design of this study.

### Patient population

We reviewed the electronic medical records of adult patients who underwent all types of lung resection requiring chest tube placement between March 2013 and August 2015. Patients who died before chest tube removal or had incomplete operation records were excluded. Patients with unclear time points of one-lung ventilation and/or two-lung ventilation in the medical records were also excluded. Patients who received pressure-controlled ventilation were also excluded because VL could not be measured as a constant value.

### Anesthesia and surgery protocol

Per routine protocol, anesthesia was induced and maintained by total intravenous anesthesia with target-controlled infusions of propofol and remifentanil. Mechanical ventilation was initiated after double-lumen endotracheal tube placement guided by fiberoptic bronchoscopy with a fraction of inspirted oxygen of 0.4 to 0.5 and a tidal volume of 7–8 mL/kg of predicted body weight with or without positive end-expiratory pressure (PEEP) of 5 to 7 cmH_2_O. During one-lung ventilation, inspired oxygen fraction of 0.5 to 1.0, a tidal volume of 5 to 6 mL/kg of predicted body weight, and PEEP of 5 to 10 cmH_2_O were applied. When the plateau airway pressure exceeded 30 cmH_2_O, the ventilation mode was switched to pressure-controlled ventilation.

Lung resection surgery was performed with either thoracoscopy or thoracotomy. After surgery, one or two 20–28 Fr chest tubes were inserted before chest wall closure and natural drainage or negative pressure suction was applied. In the general ward, chest tubes were naturally drained until its removal. Chest tubes were removed when no air bubble was visible in the water seal during coughing in a completely ambulating patient.

### Data collection

Patient demographics including age, sex, weight, height and body mass index (BMI) were collected from the electronic medical records. Previous history of pulmonary disease including tuberculosis, bronchial asthma and chronic obstructive pulmonary disease was recorded. Pulmonary function test results were collected including forced expiratory volume in 1 second (FEV1), functinoal vital capacity (FVC), their percent predicted values (%FEV1, %FVC), FEV1/FVC and diffusing capacity for carbon monoxide (DL_CO_). Surgery-related parameters including type of surgery, surgical approaches and its findings were collected by reviewing postoperative surgical records. Surgery type was categorized as major (lobectomy and bilobectomy) and minor (wedge resection and segmentectomy) lung resections. Surgical approach was classified as thoracoscopy or thoracotomy. Surgical findings including severity of pleural adhesion (none, focal, or diffuse), and the presence of pleural seeding and effusion were also recorded.

The inspiratory and expiratory tidal volumes were measured by volumeter integrated in the anesthesia machine (Primus^®^, Drägerwerk AG & Co. KGaA, Lübeck, Germany). Inspiratory and expiratory tidal volumes were collected through the data communication port of the anesthesia machine, and were automatically recorded in the anesthesia information management system at 1 minute intervals. After verifying time points according to the surgical procedures, pairs of inspiratory and expiratory tidal volumes were obtained at two different time points: during the initial two-lung ventilation before the initiation of one-lung ventilation (baseline), and during the restart of two-lung ventilation after the main surgical procedure. The median value of 5 consecutive measurements was used for analysis. VL was calculated from the pair of tidal volumes by the following equation.

$$VL\;(\%)\;=\;\frac{(inspiratory\;tidal\;volume-expiratory\;tidal\;volume)\times 100}{inspiratory\;tidal\;volume\;}$$

The duration of chest tube placement was considered identical to the duration of air leakage and measured as POD (days). The primary outcome variable was PAL, which was defined when the chest tube was maintained beyond POD 5 in accordance with the Society of Thoracic Surgeons database definition [3,6]. Patients were classified as no PAL group or PAL group depending on the presence of persistent air leak beyond POD 5.

The durations of surgery, anesthesia and one-lung ventilation, and postoperative hospital stay were also recorded.

### Sample size calculation

The sample size was calculated to detect independent variables in Cox proportional hazards regression analysis. We assumed that the incidence of PAL in our surgical patients to be 20%, and the number of independent risk factors would be at most 10 according to previous reports. Based on the report of Peduzzi and colleagues [9], the sample size was calculated using the following formula and estimated to be 500:

N=10xnumberofpredictors/proportionofpositivecases=10x10/0.2=500

### Statistical analysis

Categorical variables were expressed as number (%) and continuous variables were expressed as mean ± SD. Characteristics of no PAL and PAL groups were compared using Pearson chi square test or Fisher’s exact test and unpaired T test.

Both continuous and binary variables of VL were tested. Continuous VL values were categorized using Youden’s J statistic, which is the criterion that best distinguished the PAL group from the no PAL group. Cox proportional hazards regression analysis was performed using VL as a predictor and PAL as the dependent outcome.

To evaluate whether VL retains its power to predict PAL after adjustments, Cox proportional hazards regression analysis was performed using previously reported risk factors in addition to VL as a potential predictor and PAL as a dependent variable. Continuous variables were converted to binary variables using Youden’s J statistic. Pulmonary function test results were converted to six binary variables according to the cutoffs used in the previous studies to predict PAL: FEV1 <80% [6], FEL1 <1.5 L [10], both FEV1 and FVC <70% [11], FEV1 <35% [12], FEV1/FVC <50% [5], and DL_CO_ <80% [10]. Multivariable Cox proportional hazards regression analyses were performed with a stepwise forward variable selection. The model assumption was tested with log minus log survival plots and Schoenfeld residuals. C-statistic of the final model was calculated using the model-predicted propensity scores, and compared with that of a recent risk score model by Pompili and colleagues [13]. The followings are the formulas to calculate the risk scores of Pompili and colleagues and ours. Risk score was calculated by giving 1 if the condition in the parenthesis is correct, 0 if not.

- Risk score of Pompili and colleagues’ study = (Male) + (FEV1 < 80%) + 2×(BMI < 18.5 kg/m^2^)
- Risk score of our study = 1.59×(ventilatory leak > 9.5%) + 1.21×(Male) + 1.22×(Age older than 60) + 1.47×(BMI < 21.5 kg/m^2^) + 1.71×(FEV1 < 80%) + 1.56×(thoracotomy) + 1.80×(major lung resection) + 1.95×(One lung ventilation time > 2.1 hour)

SPSS software (version 21.0, IBM Corp., Armonk, NY, USA) and MedCalc software (version 16.0, www.medcalc.org, Mariakerke, Belgium) were used for statistical analyses. A *P*-value <0.05 was considered significant.

## Results

**Fig 1:**
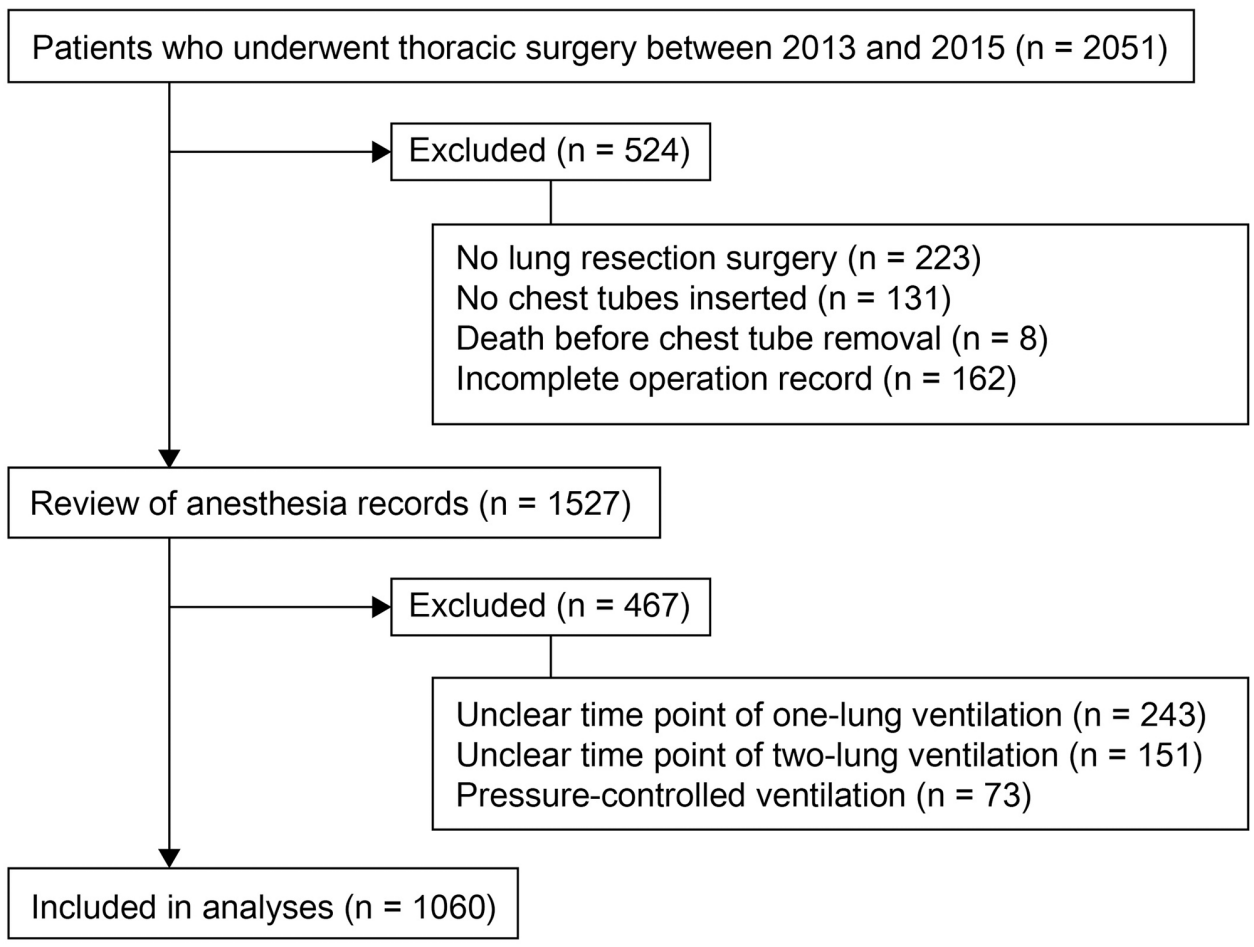
Flow diagram of study.

Fig 1 shows the flow diagram outlining included and excluded cases according to the study design. Among the 2051 patients who underwent thoracic surgery between 2013 and 2015, a total of 1527 patients’ anesthesia records were reviewed and 1060 patients were included in the regression analysis.

The median duration of chest tube placement was 3 days and the incidence of PAL was 18.7% (198/1060). Differences between no PAL and PAL groups are described in Table 1.

**Table 1:** Patient characteristics.

Characteristic	No prolonged air leak (n = 862)	Prolonged air leak (n = 198)	*P*-value
**Age (years)**	59 ± 14	63 ± 12	<0.001
**Gender (Male/Female)**	476/386	150/48	<0.001
**Weight (kg)**	63 ± 10	61 ± 10	0.018
**Height (cm)**	163 ± 9	165 ± 8	0.018
**BMI (kg/m**^**2**^**)**	23.4 ± 3.2	22.3 ± 3.2	<0.001
**Premedical pulmonary disease**			
** Tuberculosis**	56 (6.5%)	20 (10.1%)	0.105
** Asthma**	10 (1.2%)	3 (1.5%)	0.959
** Chronic obstructive pulmonary disease**	182 (21.1%)	89 (44.9%)	<0.001
**Pulmonary function test**			
** FEV1 (L)**	2.6 ± 0.69	2.4 ± 0.6	0.004
** %FEV1 (%)**	103 ± 18	93 ± 17	<0.001
** FVC (L)**	3.5 ± 0.9	3.5 ± 0.8	0.250
** %FVC (%)**	100 ± 15	96 ± 15	0.004
** FEV1/FVC (%)**	75 ± 9	70 ± 13	<0.001
** DL**_**CO**_ **(mL/mmHg/min)**	18 ± 5	17 ± 5	0.597
** DL**_**CO**_ **(%)**	96 ± 19	94 ± 19	0.501
**Duration of surgery (min)**	124 ± 63	189 ± 76	<0.001
**Duration of one lung ventilation (min)**	105 ± 55	159 ± 60	<0.001
**Chest tube in situ (days)**	3 ± 1	8 ± 3	<0.001
**Postoperative hospital length of stay (days)**	4 ± 2	10 ± 5	<0.001
**Operative information**			
** Emergency**	84 (9.7%)	19 (9.6%)	1.000
** Thoracotomy**	42 (4.9%)	31 (15.7%)	<0.001
** Major lung resection**	399 (46.3%)	148 (74.7%)	<0.001
** Pleural adhesion**			<0.001
** Focal**	278 (32.3%)	72 (36.4%)	
** Diffuse**	107 (12.4%)	52 (26.3%)	
** Effusion**	57 (6.6%)	20 (10.1%)	0.120
** Seeding**	16 (1.9%)	2 (1.0%)	0.599
**Ventilatory information**			
** Baseline**			
** Inspiratory tidal volume (mL)**	449 ± 67	455 ± 60	0.192
** Expiratory tidal volume (mL)**	442 ± 68	449 ± 61	0.156
Leak (%)*	1.4 ± 1.5	1.2 ± 1.3	0.322
** Restart of two-lung ventilation**			
** Inspiratory tidal volume (mL)**	452 ± 64	470 ± 64	<0.001
** Expiratory tidal volume (mL)**	410 ± 65	395 ± 75	0.010
Leak (%)*	9.2 ± 7.7	15.7 ± 12.7	<0.001

Data are mean ± SD or number (%).

* Ventilatory leak was calculated as (inspiratory tidal volume—expiratory tidal volume)/ inspiratory tidal volume x 100 (%)

BMI = body mass index, DL_CO_ = diffusing capacity for carbon monoxide, FEV1 = forced expiratory volume in 1 second, FVC = functional vital capacity.

**Fig 2:**
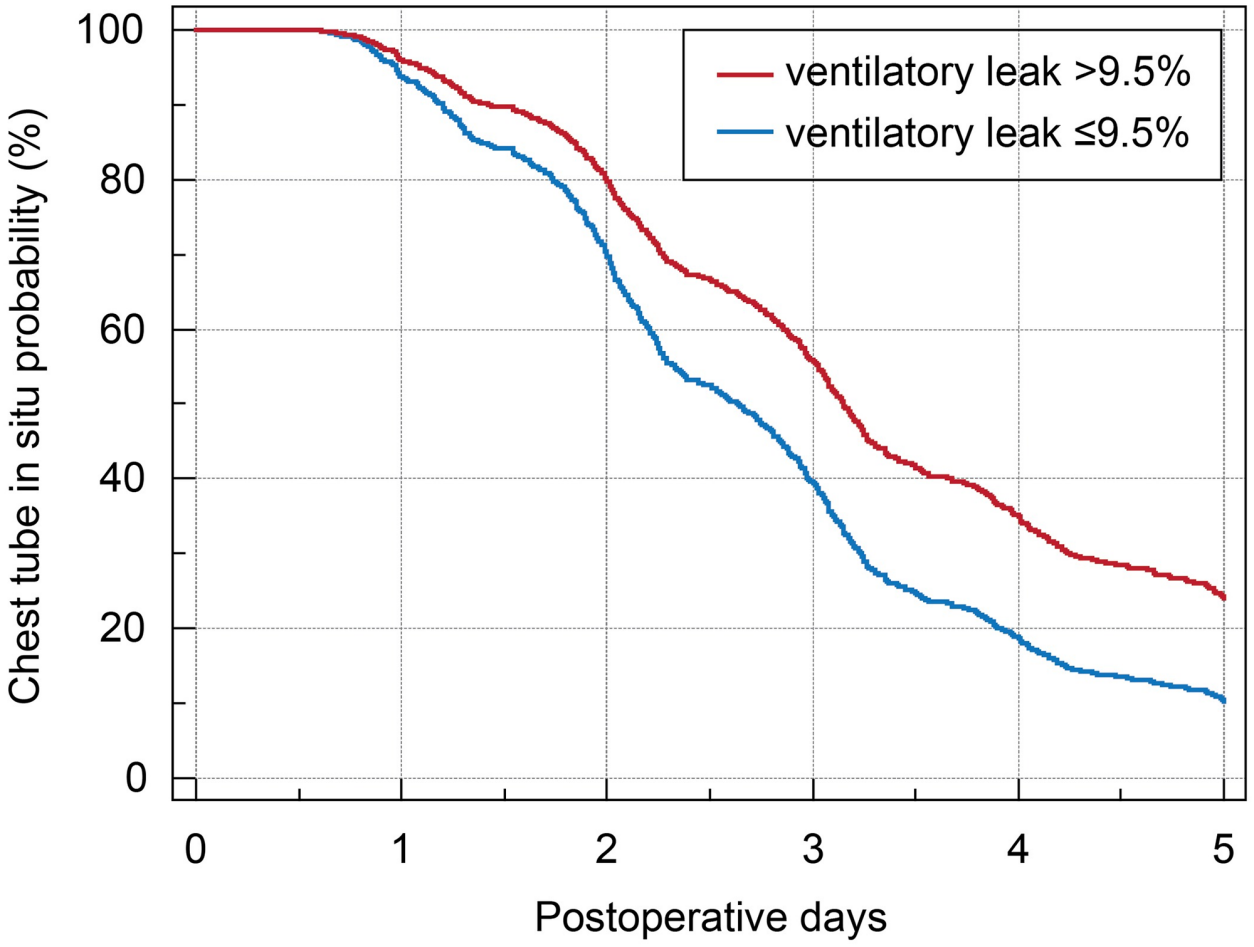
The probabilities of chest tube in situ in ventilatory leak >9.5% and ventilatory leak ≤9.5% groups. The probability of chest tube in situ is plotted during the postoperative days according to the categorical variable of the ventilatory leak subgroups after adjusting for all other covariates in the full model. The ventilatory leak >9.5% group has an increased risk of prolonged air leak, which was defined as chest tube in situ over postoperative day 5, compared to the ventilatory leak ≤9.5% group.

The binary criteria of continuous variables were determined as follows: VL >9.5%, age >60 years, BMI <21.5 kg/m^2^, and duration of one-lung ventilation >2.1 hours. Univariable Cox proportional hazards regression analysis showed that odds ratios of VL to predict PAL were 1.05 (95% CI, 1.04–1.06, *P* <0.001) per % increase of VL, or 1.89 (95% CI, 1.64–2.18, *P* <0.001) when VL was greater than 9.5%. Cox proportional hazards regression analysis identified 8 independent risk factors of PAL (Table 2). After adjustment, the VL remained a significant predictor of PAL with odds ratios of 1.03 (95% CI, 1.02–1.04, *P* <0.001) as a continuous variable or 1.59 (95% CI, 1.37–1.85, *P* <0.001) as a binary variable (VL >9.5%). Fig 2 shows that the risk of chest tube in situ until POD 5 is significantly higher in the patients with VL >9.5% after adjustments of other risk factors.

**Table 2:** Predictors of prolonged air leak after lung resection.

Variables	Adjusted odds ratio (95% CI)	*P*-value
**Ventilatory leak >9.5%**	1.59 (1.37–1.85)*	<0.001
**Male gender**	1.21 (1.05–1.39)	0.010
**Age >60 years**	1.22 (1.06–1.40)	0.007
**BMI <21.5 kg/m**^**2**^	1.47 (1.25–1.73)	<0.001
**FEV1 <80%**	1.71 (1.29–2.26)	<0.001
**Thoracotomy**	1.56 (1.13–2.15)	0.006
**Major lung resection**	1.80 (1.56–2.08)	<0.001
**One lung ventilation >2.1 hours**	1.95 (1.67–2.29)	<0.001

* The adjusted odds ratio of ventilatory leak per % increase of leak was 1.03 (95% CI, 1.02–1.04, *P* <0.001).

BMI = body mass index, FEV1 = forced expiratory volume in 1 second, FVC = functional vital capacity.

**Fig 3:**
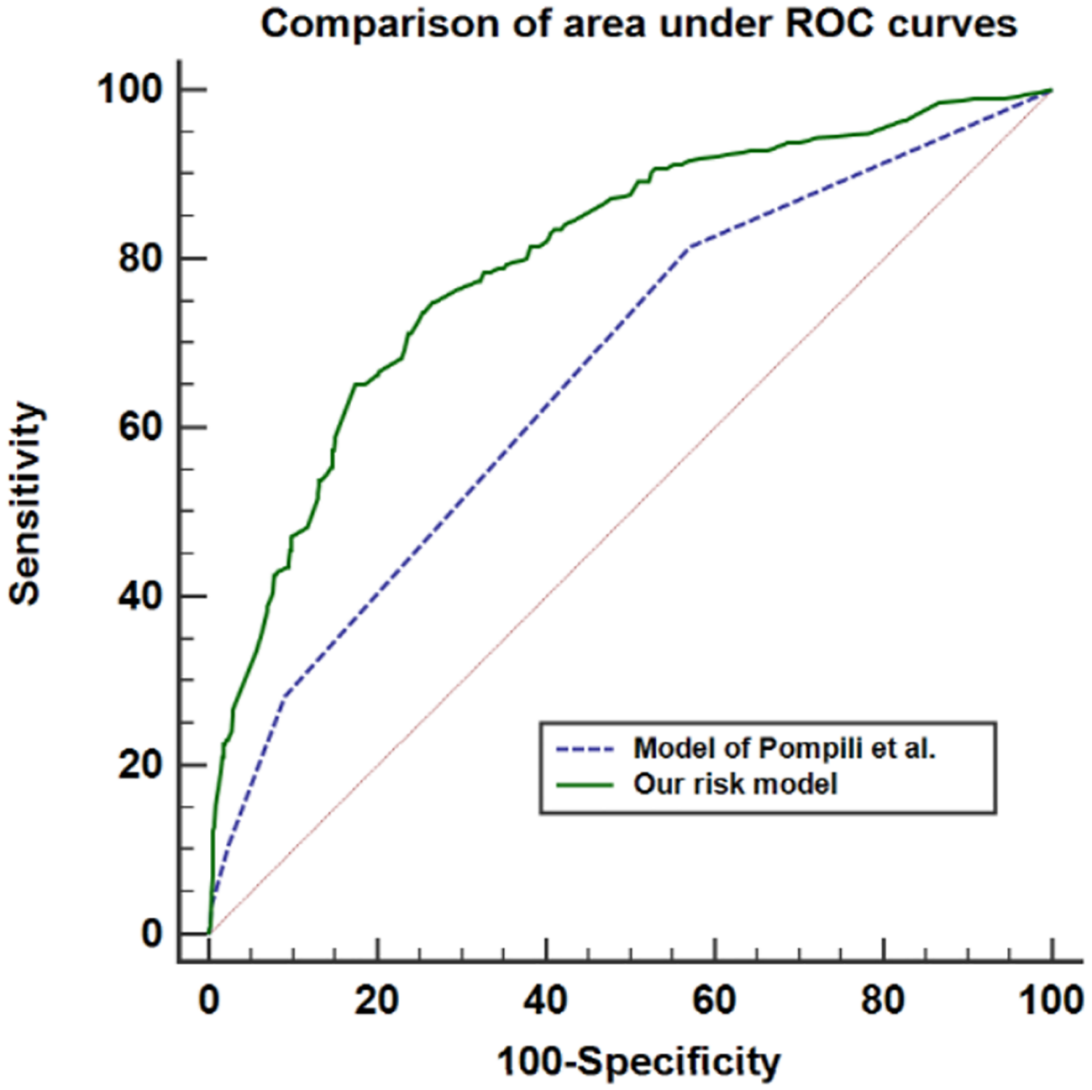
Comparison of areas under the receiver operating characteristic curves. Area under curve of the previous model [13] was significantly smaller than that of the new model (0.67, 95% CI 0.64–0.70 vs 0.80, 95% CI 0.77–0.82; *P* <0.0001).

The C-statistic of the new model having 8 predictors was 0.80 (95% CI, 0.77–0.82), which was significantly larger than that of the previous model by Pompili and colleagues (0.67, 95% CI 0.64–0.70; *P* <0.001) (Fig 3).

## Discussion

In the present study, intraoperative VL was quantitatively measured immediatley after lung parenchymal resection and identified as an independent predictor of PAL.

A proposed pathogenesis of PAL after lung resection surgery is that the dissected lung surface becomes separated from the pleura and fails to adequately heal [14]. Preoperative pulmonary function test results suggestive of chronic obstructive pulmonary disease have been consitently identified as a risk factor of PAL. In our study, all variables of pulmonary function test were tested and FEV1 <80% was found to be an independent predictor among preoperative risk factors. The reduced pulmonary function was regarded as a reflection of pathologic parenchymal change. We also observed a statistically significant increase in risk in patients over the age of 60 years. Old age was reported to be an important risk factor for PAL [6]. Elderly patients are more likely to have fragile lung parenchyma with reduced healing capacity, which may lead to PAL. Low BMI was also a significant risk factor of PAL, possibly because it represents poor nutritional status, which may also contribute to delayed healing [6,15]. Reduced preoperative pulmonary function, old age, and low BMI are consistent with the factors included in the previously reported scoring system to predict PAL [6]. Sex was not a significant risk factor in previous studies [5,11], however, two recent reports found the male sex as a risk factor [16,17]. These preoperative risk factors constitute baseline risks for PAL that are not affected by the surgery itself.

Intraoperative risk factors of PAL are considered to be more closely linked to the immediate and prolonged postoperative air leak. Intraoperative risk factors can be modified during lung resection and reassessed at the end of surgery. Among the intraoperative variables, the most potent predictor for PAL in terms of odds ratio was the duration of one-lung ventilation, which was not previously reported. Prolonged one-lung ventilation may be considered as a comprehensive indicator of surgery-related parameters including pleural adhesion [6,16,18], extent of surgery [18–20], and proficiency of the surgeon [2]. In accordance with previous reports [5,16,17,21,22], lobar or bilobar resection, compared to minor resection, was identified as a risk factor of PAL in our study. However, in contrast to previous reports, open thoracotomy was identified as an independent risk factor for PAL. Open thoracotomy carries the risk of pleural and parenchymal lung injury due to excessive surgical traction and blind exploration of deep structures [19].

In contrast to preoperative and abovementioned intraoperative risk factors that cannot be modified during surgery, VL may be reduced with additional intraoperative interventions. Because most of the postoperative therapeutic options for PAL have weak supporting evidence [3], a surgical strategy may be more effective in reducing air leaks. Pleural tent, pneumoperitonium, surgical sealants, staple-line buttressing, and tissue transposition have been suggested as surgical techniques for treatment of extreme air leak during pulmonary resection [8,23–27]. However, routine use of these techniques is not recommended because they are costly and time consuming. In order to determine whether additional surgical intervention should be used, air leakage should be assessed objectively and quantitatively, but no such method has been proposed. The traditional method of evaluating air leakage during surgery is to fill the thoracic cavity with saline and observe the degree of air bubbles generated during manual positive pressure ventilation. A simple four-point scale has been proposed, but it is still subjective and depends on the individual surgeon’s experience [8]. In addition, qualitative assessment of air leak by observing insufficient filling of ventilator bellows or bag of the anesthesia machine despite considerable fresh gas flow rate has been widely used in clinical practice. Our method of detecting intraoperative VL uses flowmeter attached to modern anesthesia machines to accurately quantify air leaks without interrupting mechanical ventilation.

We may suggest several practical recommendations to reduce postoperative PAL based on our study results. First, more caution should be taken in patients with baseline risk factors such as male sex, low BMI, and impaired pulmonary function. Second, surgical modification may be considered for open thoracotomy, major lung resection, and anticipated prolonged operation time. Finally, monitoring of intraoperative VL is recommended to provide real-time intraoperative guidance to determine whether surgical adjuncts are needed or simple repair would be sufficient to reduce postoperative air leak.

The results of our study should be interpreted cautiously with regards to the following limitations. First, our study is a retrospective observational study and unknown bias associated with the study design may have affected our analysis. There may be different causes of prolonged chest tube duration other than air leaks in our study population. However, most of our risk factors are consistent with previously reported PAL risk factors, which suggests that most cases are likely to be related to air leaks. Second, more than half of the patients initially assessed were excluded, thus the validity of our study results may be limited. Nonetheless, we still believe that reduced bias by prudent inclusion criteria of our study has led to reliable conclusions. Third, recent lung-protective ventilatory strategies employ small tidal volume, large PEEP and pressure-controlled ventilation, which are different from our anesthesia protocol [28,29]. Since VL may be modified by use of small tidal volume and/or large PEEP, our VL criteria should be applied with caution when performing anesthesia according to the lung-protective strategy. Prospective studies will be needed to evaluate the usefulness of VL assessment in lung-protective ventilation.

In conclusion, we demonstrated that VL at the end of lung parenchymal resection is an objective and quantitative measurement of air leakage during lung resection and may be an independent predictor of PAL. The VL measurement during surgery may be useful for deciding whether or not to use surgical adjuncts for the treatment of air leak during pulmonary resection and evaluating the effect of the treatment immediately. A prospective study is required to confirm the impact of VL-guided surgical repair or use of surgical adjuncts on the reduction of the incidence of PAL.

## Supporting information

S1 Checklist A STROBE checklist for the present study. (DOC)

S1 File A dataset for the present study. (XLSX)
